# Erythematous plaques on the bilateral dorsal hands

**DOI:** 10.1002/ski2.286

**Published:** 2023-09-10

**Authors:** Mohammad Fardos, Benjamin R. Cooper, Sara Holt, David Esguerra

**Affiliations:** ^1^ HCA Healthcare/USF Morsani College of Medicine GME Programs Largo Medical Center Largo Florida USA; ^2^ HCA Florida Orange Park Hospital Orange Park Florida USA

## Abstract

Neutrophilic dermatosis of the dorsal hands is a rare cutaneous disorder characterized by the sudden onset of painful, haemorrhagic, and pustular bullae with violaceous rims on the dorsal aspects of the hands. Here we present a 61‐year old male with a 1‐week history of tender, haemorrhagic, and pustular bullae on both dorsal hands, evolving into ulcerative plaques with haemorrhagic crusting over 5 days. A 4 mm lesional punch biopsy revealed a diffuse dermal infiltrate of numerous neutrophils intermixed with lymphocytes and histiocytes, along with epidermal spongiosis and neutrophilic exocytosis. No leukocytoclastic vasculitis was identified. The provided images document the progression of this rare inflammatory process. Understanding such cases is crucial for accurate diagnosis and management.

## Graphical abstract

This report documents the clinical and histopathologic diagnosis of neutrophilic dermatosis of the dorsal hands. The images provided showcase the evolution of this rare inflammatory process.

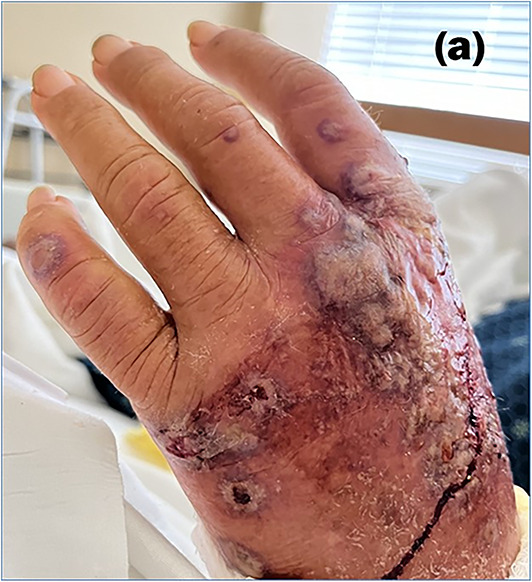

A 61‐year‐old male presented with a 1‐week history of tender haemorrhagic and pustular bullae with violaceous rims involving the dorsal aspects of both hands (Figure 1a). The lesions evolved into ulcerative plaques with haemorrhagic crusting 5 days later (Figure 1b). A 4 mm lesional punch biopsy was performed, revealing a diffuse dermal infiltrate of numerous neutrophils intermixed with lymphocytes and histiocytes, as well as epidermal spongiosis and neutrophilic exocytosis (Figure 1c,d). No leukocytoclastic vasculitis was identified. Direct immunofluorescence was non‐specific and showed no vascular deposition of immunoglobulins, fibrinogen, or complement. The clinicopathologic correlation supported a diagnosis of neutrophilic dermatosis of the dorsal hands.^1^, ^2^ Investigation for underlying diseases like malignancy and inflammatory bowel disease was initiated, however, none were found as patient was lost to follow‐up.

**FIGURE 1:**
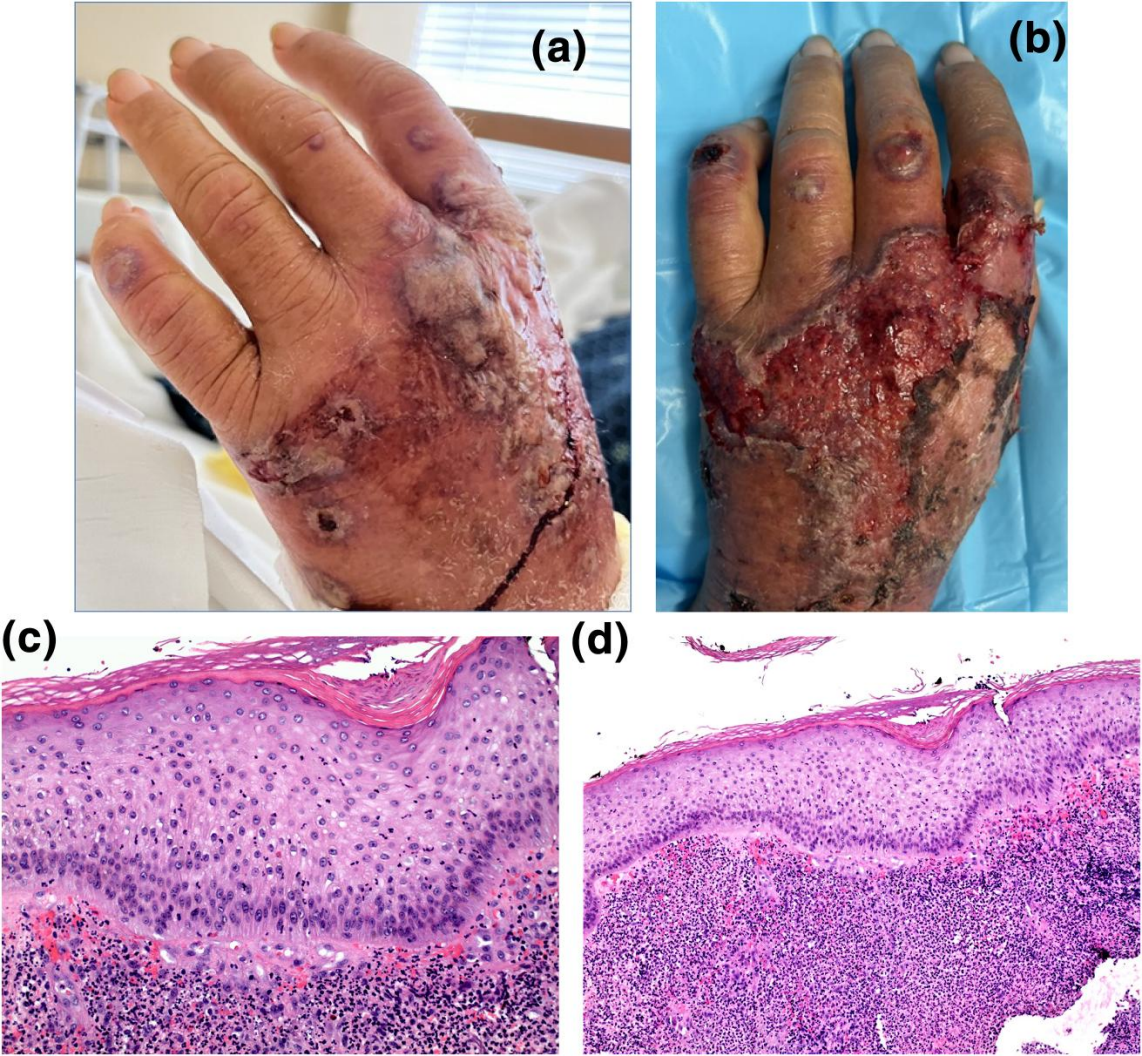
Multiple tender haemorrhagic and pustular bullae with violaceous rims involving the dorsal aspect of the left hand. (a) Multiple tender haemorrhagic and pustular bullae with violaceous rims involving the dorsal aspect of the left hand. (b) Ulcerative plaques with haemorrhagic crusting involving the dorsal aspect of the left hand. (c) At 20x magnification, a diffuse dermal infiltrate of numerous neutrophils intermixed with lymphocytes and histiocytes, as well as epidermal spongiosis and neutrophilic exocytosis is seen. No leukocytoclastic vasculitis was identified. (d) At 10x magnification, a diffuse dermal infiltrate of numerous neutrophils intermixed with lymphocytes and histiocytes, as well as epidermal spongiosis and neutrophilic exocytosis is seen.

## AUTHOR CONTRIBUTIONS

**Mohammad Fardos**: Conceptualization (equal); writing—original draft (lead); writing—review & editing (equal). **Benjamin R. Cooper**: Writing—original draft (equal); writing—review & editing (equal). **Sara Holt**: Supervision (equal); writing—original draft (equal); writing—review & editing (equal). **David Esguerra**: Data curation (equal); supervision (equal).

## CONFLICT OF INTEREST STATEMENT

None to declare.

## ETHICS STATEMENT

Not applicable.

## Data Availability

Data sharing is not applicable to this article as no new data were created or analysed in this study.
